# The crystal structures and Hirshfeld surface analyses of a cadmium(II) and a zinc(II) mononuclear complex of the new tetrakis-substituted pyrazine ligand *N*,*N*′,*N*′′,*N*′′′-[pyrazine-2,3,5,6-tetra­yltetra­kis­(methyl­ene)]tetra­kis­(*N*-methyl­aniline)

**DOI:** 10.1107/S2056989020001644

**Published:** 2020-02-18

**Authors:** Ana Tesouro Vallina, Helen Stoeckli-Evans

**Affiliations:** a Institute of Chemistry, University of Neuchâtel, Av. de Bellevaux 51, CH-2000 Neuchâtel, Switzerland; b Institute of Physics, University of Neuchâtel, rue Emile-Argand 11, CH-2000 Neuchâtel, Switzerland

**Keywords:** crystal structure, tetra­kis-substituted pyrazine, cadmium(II), zinc(II), mononuclear complexes, C—H⋯π inter­actions, metal–halide⋯π(pyrazine) contacts

## Abstract

The whole mol­ecule of the cadmium(II) complex, di­iodido­{*N*,*N*′,*N*′′,*N*′′′-[pyrazine-2,3,5,6-tetra­yltetra­kis­(methyl­ene)]tetra­kis­(*N*-methyl­aniline)-κ^3^ *N* ^2^,*N* ^1^,*N* ^6^}cadmium(II), [CdI_2_(C_36_H_40_N_6_)], (**I**), of the ligand *N*,*N*′,*N*′′,*N*′′′-[pyrazine-2,3,5,6-tetra­yltetra­kis­(methyl­ene)]tetra­kis­(*N*-methyl­aniline) (**L**), is generated by a twofold rotation symmetry; the twofold axis bis­ects the cadmium atom and the nitro­gen atoms of the pyrazine ring. The ligand coordinates in a mono-tridentate manner and the cadmium atom has a fivefold CdN_3_I_2_ coordination environment with a distorted shape. In the zinc(II) complex, dichlorido{*N*,*N*′,*N*′′,*N*′′′-[pyrazine-2,3,5,6-tetra­yltetra­kis­(methyl­ene)]tetra­kis­(*N*-methyl­aniline)-κ^3^ *N* ^2^,*N* ^1^,*N* ^6^}zinc(II) di­chloro­methane 0.6-solvate, [ZnCl_2_(C_36_H_40_N_6_)]·0.6CH_2_Cl_2_, (**II**), ligand **L** also coordinates in a mono-tridentate manner and the zinc atom has a fivefold ZnN_3_Cl_2_ coordination environment with a distorted shape. It crystallized as a partial di­chloro­methane solvate. In the crystal of **I**, the complex mol­ecules are linked by weak C—H⋯I contacts, forming ribbons propagating along [100]. In the crystal of **II**, the complex mol­ecules are linked by a series of C—H⋯π inter­actions, forming layers lying parallel to the (11) plane. In the crystals of both compounds there are metal–halide⋯π(pyrazine) contacts present. The Hirshfeld analyses confirm the importance of the C—H⋯halide contacts in the crystal packing of both compounds.

## Abstract 2

In the cadmium(II) and zinc(II) complexes of the tetra­kis-substituted pyrazine ligand, *N*,*N*′,*N*′′,*N*′′′-[pyrazine-2,3,5,6-tetra­yltetra­kis­(methyl­ene)]tetra­kis­(*N*-methyl­aniline), the ligand coordinates in a mono-tridentate fashion, and both metal atoms have fivefold coordination spheres with distorted shapes.

## Chemical context   

The title ligand, *N*,*N*′,*N*′′,*N*′′′-[pyrazine-2,3,5,6-tetra­yltetra­kis(methyl­ene)]tetra­kis­(*N*-methyl­aniline) (**L**), whose synthesis and crystal structure have been described in the preceding publication (Tesouro Vallina & Stoeckli-Evans, 2020 ▸), is a new tetra­kis-substituted pyrazine derivative. It was designed to study its coordination behaviour with transition metals (Tesouro Vallina, 2001 ▸). The reaction of the ligand with CdI_2_ and ZnCl_2_ lead to the formation of the title mononuclear complexes **I** and **II**. Herein, we describe their syntheses, mol­ecular and crystal structures and the analyses of their Hirshfeld surfaces.

## Structural commentary   

**Figure 1:**
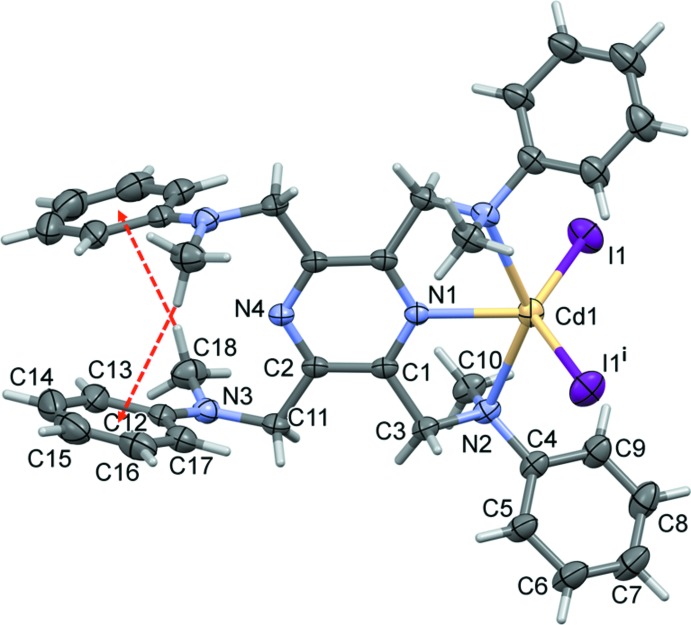
A view of the mol­ecular structure of complex **I**, with atom labelling [symmetry code (i): −*x* + 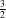, *y*, −*z*]. Displacement ellipsoids are drawn at the 30% probability level. The intra­molecular C—H⋯π inter­actions are shown as dashed red arrows (Table 2 ▸).

**Figure 2:**
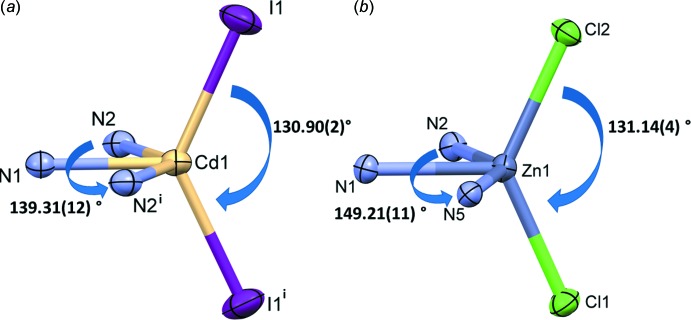
A comparison of the coordination spheres of (*a*) the cadmium atom in complex **I** [symmetry code (i): −*x* + 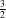, *y*, −*z*], and (*b*) the zinc atom in complex **II**.

The mol­ecular structure of the cadmium(II) complex, Cd(**L**)I_2_ (**I**), of the ligand *N*,*N*′,*N*′′,*N*′′′-[pyrazine-2,3,5,6-tetra­yltetra­kis­(methyl­ene)]tetra­kis­(*N*-methyl­aniline) (**L)**, is illustrated in Fig. 1 ▸. Selected geometrical parameters are given in Table 1 ▸. The complex possesses twofold rotation symmetry, with the twofold axis bis­ecting the cadmium atom, Cd1, and the nitro­gen atoms N1 and N4 of the pyrazine ring. The ligand coordinates in a mono-tridentate manner and the cadmium atom has a fivefold CdN_3_I_2_ coordination environment with a distorted shape (see Fig. 2 ▸ *a*). The τ_5_ parameter for the fivefold coordination of atom Cd1 is 0.14 (τ_5_ = 0 for a perfect square-pyramidal geometry and = 1 for a trigonal–pyramidal geometry; Addison *et al.*, 1984 ▸).
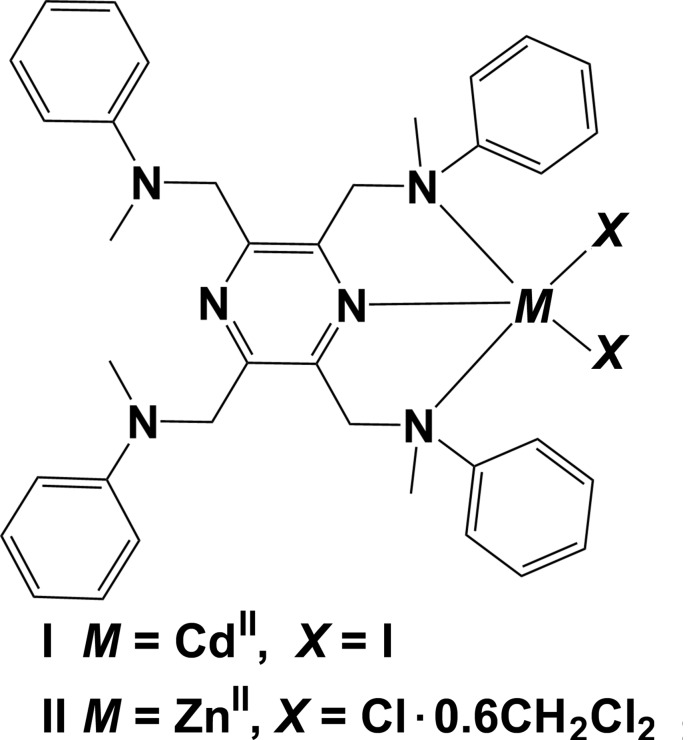


A search of the Cambridge Structural Database (CSD, Version 5.41, last update November 2019; Groom *et al.*, 2016 ▸) for a CdN_3_I_2_ coordination environment involving a pyrazine N atom yielded only one relevant structure, the CdI_2_ mononuclear complex of the ligand 2,3,5,6-tetra­kis­(pyridin-2-yl)pyrazine (TPPZ), *viz*. complex (2,3,5,6-tetra­kis­(pyridin-2-yl)pyrazine)­bis­(iodo)­cadmium(II) (GAHRIT; Saghatforoush, 2015 ▸). Here the τ_5_ parameter for the cadmium atom is 0.04. The Cd—N_pz_ bond length is *ca* 2.388 Å compared to 2.295 (3) Å in **I**, while the Cd—I bond lengths are *ca* 2.741 and 2.727 Å compared to 2.7038 (3) Å in **I**. The N-methyl­aniline groups on the non-coordinated side of the ligand are linked by intra­molecular C—H⋯π inter­actions (Fig. 1 ▸ and Table 2 ▸).

**Figure 3:**
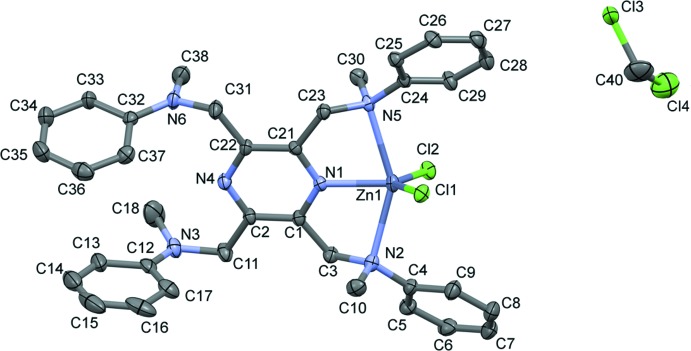
A view of the mol­ecular structure of compound **II**, with atom labelling. Displacement ellipsoids are drawn at the 30% probability level. For clarity, H atoms have been omitted.

The mol­ecular structure of the zinc(II) complex, Zn(**L**)Cl_2_·0.6(CH**_2_**Cl_2_) (**II**), is illustrated in Fig. 3 ▸. It crystallized as a partial di­chloro­methane solvate. Selected geom­etrical parameters are given in Table 3 ▸. The ligand **L** coordinates in a mono-tridentate manner and the zinc atom, Zn1, has a fivefold ZnN_3_Cl_2_ coordination environment with a distorted shape (see Fig. 2 ▸ *b*). The τ_5_ parameter for atom Zn1 is 0.30.

A search of the CSD for a ZnN_3_Cl_2_ coordination environment involving a pyrazine N atom yielded five relevant structures, which again involve the ligand TPPZ. They include two polymorphs of the mononuclear complex di­chloro-[2,3,5,6-tetra­kis­(2-pyrid­yl)pyrazine-*N*,*N*′,*N*′′]zinc(II): a monoclinic polymorph (WAGPOJ; Graf *et al.*, 1993 ▸) and a triclinic polymorph (WAGPOJ01; Saljooghi & Fatemi, 2011 ▸). There are two structures of the binuclear complex [μ_2_-2,3,5,6-tetra­kis­(2-pyrid­yl)pyrazine]­tetra­chloro­dizinc(II): one hydrated (DOMHOD; Trivedi *et al.*, 2009 ▸), the other not (PAPCER; Hong *et al.*, 2017 ▸), and finally, the unusual polynuclear complex octa­kis­(μ_2_-chloro)­bis­[μ_2_-2,3,5,6-tetra­kis­(2-pyrid­yl)pyrazine]­dodeca­chloro­tetra­aqua­deca­zinc (WIBVOS; Graf & Stoeckli-Evans, 1994 ▸). For these five structures, the τ_5_ parameter for the zinc atoms varies from 0.08 in WAGPOJ to 0.36 in WAGPOJ01. The latter is similar to the value of 0.30 for **II**. The Zn—N_pz_ bond lengths vary from *ca* 2.141 to 2.200 Å compared to 2.057 (3) Å in **II**, while the Zn—Cl bond lengths vary from *ca* 2.232 to 2.343 Å compared to 2.2251 (10) and 2.2425 (11) Å in **II**.

**Figure 4:**
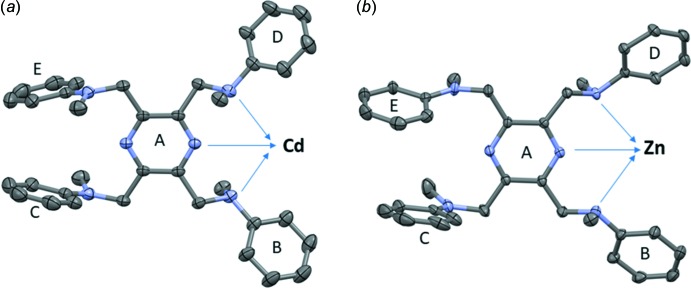
A comparison of the conformation of the ligand **L** in complexes **I** and **II**. For complex **I**, which possesses twofold rotation symmetry, ring *D* = *B* ^i^, and ring *E* = *C* ^i^ [symmetry code: (i) −*x* + 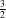, *y*, −*z*].

The conformation of the ligand **L** differs in the two complexes (Fig. 4 ▸). The orientation of the phenyl rings with respect to the pyrazine ring and to each other is slightly different, and the various dihedral angles are compared in Table 5 ▸. It can be seen that the most significant difference, of 20.9 (2)°, involves the orientation of ring *D* (ring *B* ^i^ in **I**) with respect to ring *E* (ring *C* ^i^ in **I**).

## Supra­molecular features   

**Figure 5:**
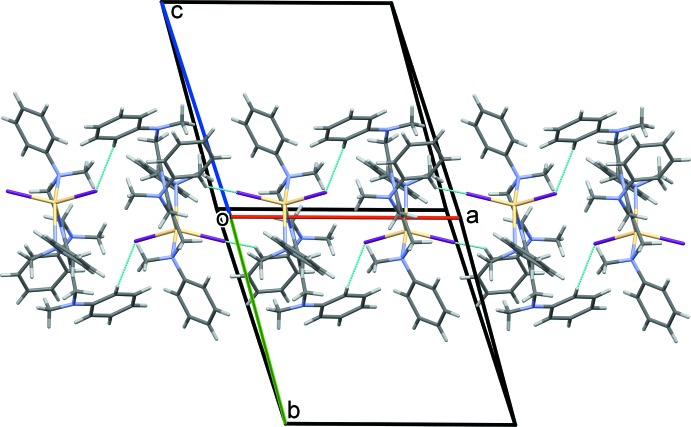
A view normal to plane (011) of the crystal packing of complex **I**. The weak C—H⋯I inter­actions are shown as dashed lines (Table 2 ▸).

**Figure 6:**
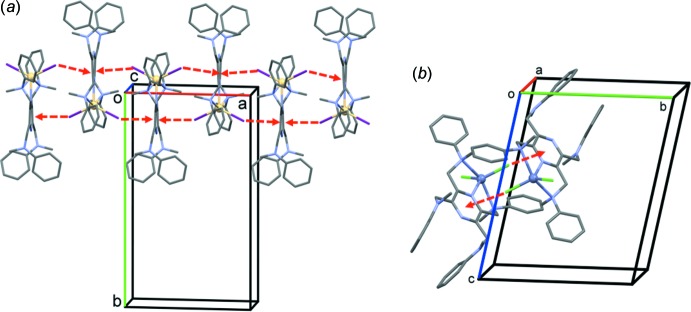
(*a*) A partial view along the *c* axis of the crystal packing of **I**, showing the Cd—I⋯π(pyrazine) inter­actions (Table 2 ▸; dashed red arrows), (*b*) a partial view along the *a* axis of the crystal packing of **II**, showing the Zn—Cl⋯π(pyrazine) inter­actions (Table 4 ▸; dashed red arrows). For clarity, the di­chloro­methane mol­ecule has been omitted.

A partial view of the crystal packing of **I** is shown in Fig. 5 ▸. Mol­ecules are linked by weak C—H⋯I contacts, forming ribbons propagating along [100]; see Table 2 ▸. There are Cd—I⋯π(pyrazine) contacts present, consolidating the chains propagating along the *a-*axis direction (Fig. 6 ▸ *a* and Table 2 ▸). This situation is similar to that observed in the crystal of the CdI_2_ complex of TPPZ (GAHRIT; Saghatforoush, 2015 ▸). There, the I⋯centroid(pyrazine ring) distance is 3.699 (1) Å with a Cd—I⋯centroid angle of 175.92 (12)°, compared to 3.9593 (12) Å and 155.19 (3)° in complex **I** (Fig. 6 ▸ *a* and Table 2 ▸).

**Figure 7:**
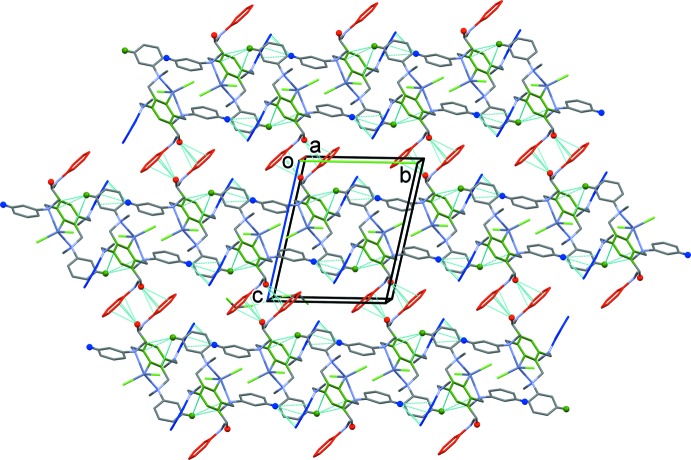
A view along the *a* axis of the crystal packing of compound **II**. The various C—H⋯π inter­actions (Table 4 ▸; blue, red and green) are shown as dashed lines. The di­chloro­methane mol­ecule has been omitted, and only the H atoms (blue, red and green) involved in the C—H⋯π inter­actions have been included.

In the crystal of **II**, mol­ecules are linked by a series of C—H⋯π inter­actions, forming layers lying parallel to the (1
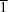
1) plane; see Fig. 7 ▸ and Table 4 ▸. The di­chloro­methane mol­ecules are linked across a center of symmetry with a short Cl4⋯Cl4(−*x*, −*y*, −z + *2*) contact of 3.045 (5) Å and do not participate in any significant inter­molecular inter­actions with the complex mol­ecule. There are Zn—Cl⋯π(pyrazine) contacts present, which link inversion-related mol­ecules, forming dimers (Fig. 6 ▸ *b* and Table 5 ▸). This arrangement is similar to that observed in the crystal structure of the ZnCl_2_ complex of TPPZ (PAPCER; Hong *et al.*, 2017 ▸). This compound crystallized with two independent mol­ecules in the asymmetric unit. There, the Cl⋯centroid(pyrazine ring) distances are *ca* 3.087 and 3.167 Å, with the corresponding Zn—Cl⋯centroid angles being *ca* 152.62 and 141.76°. In the crystal structure of WIBVOS, a similar inter­action is present with a Cl⋯centroid(pyrazine ring) distance of *ca* 3.987 Å and a Zn—Cl⋯centroid angle of *ca*. 170.96°. In complex **II**, the corresponding Cl⋯centroid(pyrazine ring) distance and Zn—Cl⋯centroid angle are 3.683 (2) Å and 155.96 (6)°, respectively (Table 4 ▸).

## Hirshfeld surface analysis and two-dimensional fingerprint plots   

The Hirshfeld surface analysis (Spackman & Jayatilaka, 2009 ▸) and the associated two-dimensional fingerprint plots (McKinnon *et al.*, 2007 ▸) were performed with *CrystalExplorer17* (Turner *et al.*, 2017 ▸). The Hirshfeld surfaces are colour-mapped with the normalized contact distance, *d*_norm_, ranging from red (distances shorter than the sum of the van der Waals radii) through white to blue (distances longer than the sum of the van der Waals radii). A summary of the short inter­molecular contacts in the crystal structures of **I** and **II** is given in Table 6 ▸.

**Figure 8:**
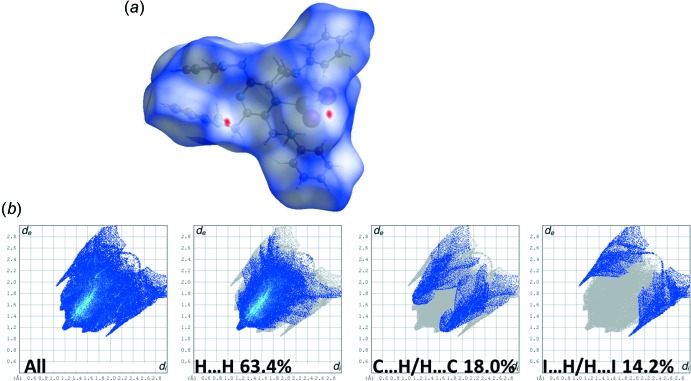
(*a*) The Hirshfeld surface of complex **I**, mapped over *d*_norm_, in the colour range −0.0713 to 1.5380 a.u., (*b*) the full two-dimensional fingerprint plot for complex **I**, and fingerprint plots delineated into H⋯H, C⋯H/H⋯C and I⋯H/H⋯I contacts.

For complex **I**, the Hirshfeld surface (HS) mapped over *d*_norm_, and the two-dimensional fingerprint plots are given in Fig. 8 ▸. The red spots on the HS (Fig. 8 ▸ *a*) correspond to the I⋯H contacts, which give a pair of spikes in the fingerprint plot (Fig. 8 ▸ *b*) at *d*_e_ + *d*_i_ ≃ 3.0 Å, contributing 14.2% to the HS. The H⋯H contacts contribute 63.4% and the C⋯H contacts 18.0%. Any other atom–atom contacts contributed less than 2% and have not been included here.

**Figure 9:**
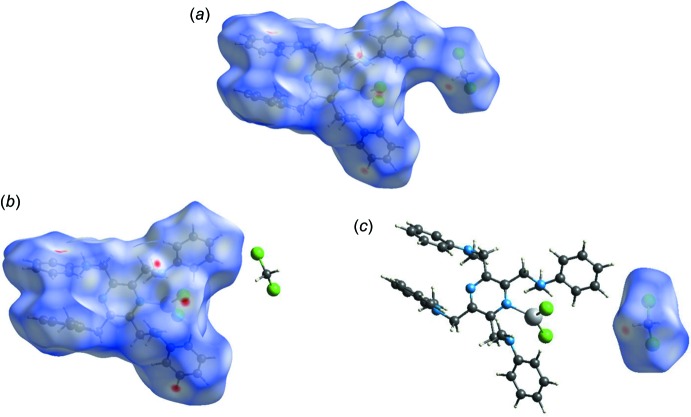
(*a*) The Hirshfeld surface of compound **II**, mapped over *d*_norm_, in the colour range −0.2597 to 1.5438 a.u., (*b*) the Hirshfeld surface of complex **II**, mapped over *d*_norm_, in the colour range −0.0933 to 1.5453 a.u., (*c*) the Hirshfeld surface of the solvent mol­ecule, mapped over *d*_norm_, in the colour range −0.2602 to 1.4344 a.u..

**Figure 10:**
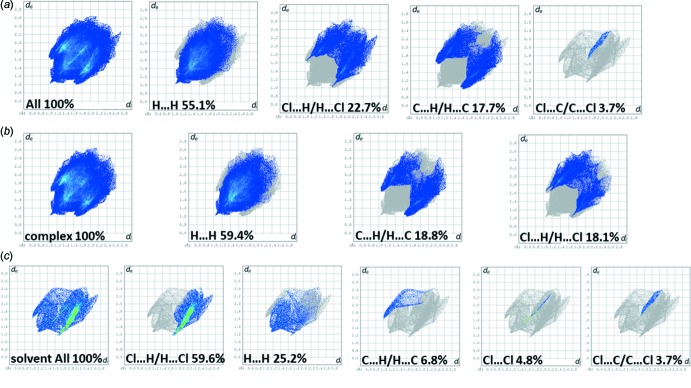
(*a*) The full two-dimensional fingerprint plot for compound **II**, and fingerprint plots delineated into H⋯H, Cl⋯H/H⋯Cl, C⋯H/H⋯C and Cl⋯C/C⋯Cl contacts, (*b*) the full two-dimensional fingerprint plot for complex **II**, and fingerprint plots delineated into H⋯H, C⋯H/H⋯C and Cl⋯H/H⋯Cl contacts, (*c*) the full two-dimensional fingerprint plot for the solvent mol­ecule and fingerprint plots delineated into Cl⋯H/H⋯Cl, H⋯H, C⋯H/H⋯C, Cl⋯Cl and Cl⋯C/C⋯Cl contacts.

For compound **II**, the Hirshfeld surface mapped over *d*_norm_, is shown in Fig. 9 ▸ *a*, and that for the complex itself and the solvent mol­ecule in Figs. 9 ▸ *b* and 9*c*, respectively. The faint red spots correspond to the Cl⋯H contacts in the crystal. These give a pair of spikes in the fingerprint plots, at *d*_e_ + *d*_i_ ≃ 2.7 Å, contributing 22.7%, in the compound (Fig. 10 ▸ *a*) and at *d*_e_ + *d*_i_ ≃ 2.7 Å, contributing 18.1%, in the complex (Fig. 10 ▸ *b*). For the solvent mol­ecule, a single sharp spike is observed (*d*_e_ + *d*_i_ ≃ 2.8 Å) with a contribution of 59.6% to the HS (Fig. 10 ▸ *c*). The H⋯H contacts contribute 55.1, 59.4 and 25.2% to the Hirshfeld surfaces of the compound, the complex and the solvent mol­ecule, respectively, while the C⋯H contributions are 17.7, 18.8 and 6.8%, respectively. Any other atom–atom contacts contributed less than 2% and have not been included here.

## Synthesis and crystallization   

The synthesis and crystal structure of the ligand, *N*,*N*′,*N*′′,*N*′′′-[pyrazine-2,3,5,6-tetra­yltetra­kis­(methyl­ene)]tetra­kis­(*N*-meth­ylaniline) **L**, have been described in the preceding publication (Tesouro Vallina & Stoeckli-Evans, 2020 ▸).

**Synthesis of the complex [Cd(L)I_2_] (I):**

About 10 ml of a very dilute CH_2_Cl_2_ solution of ligand **L** were introduced into a glass tube and layered with *ca* 2 ml of MeOH as a buffer zone. Then, 10 ml of a dilute methano­lic solution of CdI_2_ were added slowly to avoid possible mixing. The glass tube was sealed and left at room temperature. The colour of the inter­phase changed immediately to deep yellow and in hours to green. After a few days, green rod-like crystals were formed. IR (KBr pellet, cm^−1^): 2922 (*m*), 1599 (*vs*), 1507 (*s*), 1497 (*s*), 1173 (*m*), 1120 (*m*), 751 (*s*), 694 (*s*). No elemental analytical data are available.

**Synthesis of the complex [Zn(L)Cl_2_]·0.6(CH_2_Cl_2_) (II):**

To a solution of ZnCl_2_ (0.1 mmol, 0.014 g) in 5 ml of MeOH, a solution of **L** (0.05 mmol, 0.028 g, 5 ml CH_2_Cl_2_) was added. The solution was stirred at RT for 2 h without any significant colour change. The clear light-green solution obtained was filtered to avoid any impurity and allowed to evaporate slowly. After a few days, yellow rod-like crystals were obtained. IR (KBr pellet, cm^−1^): 1599 (*vs*), 1507 (*s*), 1451 (*m*), 1363 (*s*), 1257 (*m*), 1171 (*m*), 1033 (*m*), 920 (*m*), 746 (*s*), 691 (*s*). Analysis for [Zn(C_36_H_40_N_6_)Cl_2_]·0.6CH_2_Cl_2_ (743.99 g mol^−1^): calculated C 60.50, H 5.68, N 11.65%; found C 60.66, H 5.78, N 11.93%.

## Refinement   

Crystal data, data collection and structure refinement details are summarized in Table 7 ▸. The C-bound H atoms were included in calculated positions and treated as riding on their parent C atom: C—H = 0.94–0.98 Å with *U*_iso_(H) = 1.5*U*_eq_(C-meth­yl) and 1.2*U*_eq_(C) for other H atoms.

With the STOE IPDS I, a one-circle diffractometer, for the triclinic system often only 93% of the Ewald sphere is accessible. Hence, for compound **II** the _diffrn_reflns_Laue_measured_fraction_full of 0.939 is below the required minimum of 0.95. For **II**, a small number of low-angle reflections, either in the shadow of the beam-stop or with bad agreement, were omitted during the final cycles of refinement.

## Supplementary Material

Crystal structure: contains datablock(s) I, II, global. DOI: 10.1107/S2056989020001644/xi2022sup1.cif

Structure factors: contains datablock(s) I. DOI: 10.1107/S2056989020001644/xi2022Isup2.hkl

Structure factors: contains datablock(s) II. DOI: 10.1107/S2056989020001644/xi2022IIsup3.hkl

CCDC references: 1982100, 1982099

Additional supporting information:  crystallographic information; 3D view; checkCIF report

## Figures and Tables

**Table 1:** Selected geometric parameters (Å, °) for **I** [Chem scheme1]

Cd1—N1	2.295 (3)	Cd1—I1	2.7038 (3)
Cd1—N2	2.599 (3)		
			
N1—Cd1—N2	69.65 (6)	N2—Cd1—I1^i^	95.35 (6)
N2^i^—Cd1—N2	139.31 (12)	N2—Cd1—I1	101.29 (6)
N1—Cd1—I1	114.551 (10)	I1^i^—Cd1—I1	130.90 (2)

Symmetry code: (i) 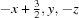.

**Table 2:** Hydrogen-bond geometry (Å, °) for **I** [Chem scheme1] C*g*3 is the centroid of the pyrazine ring N1/N4/C1/C2/C1^i^/C2^i^ and C*g*5 is the centroid of the C12–C17 ring.

*D*—H⋯*A*	*D*—H	H⋯*A*	*D*⋯*A*	*D*—H⋯*A*
C18—H18*C*⋯C*g*5^i^	0.97	2.95	3.896 (5)	165
C17—H17⋯I1^ii^	0.94	3.09	3.907 (4)	147
Cd1—I1⋯C*g* ^iii^	2.70 (1)	3.96 (1)	6.5131 (12)	155 (1)
Cd1—I1⋯C*g*3^iv^	2.70 (1)	3.96 (1)	6.5131 (12)	155 (1)

Symmetry codes: (i) 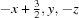; (ii) 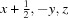; (iii) 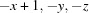; (iv) 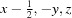.

**Table 3:** Selected geometric parameters (Å, °) for **II** [Chem scheme1]

Zn1—N1	2.057 (3)	Zn1—Cl1	2.2251 (10)
Zn1—N2	2.385 (3)	Zn1—Cl2	2.2425 (11)
Zn1—N5	2.413 (3)		
			
N1—Zn1—N2	75.02 (12)	Cl1—Zn1—N2	98.12 (8)
N1—Zn1—N5	74.23 (12)	Cl2—Zn1—N2	95.70 (9)
N2—Zn1—N5	149.21 (11)	Cl1—Zn1—N5	93.02 (7)
N1—Zn1—Cl1	114.15 (9)	Cl2—Zn1—N5	98.34 (8)
N1—Zn1—Cl2	114.68 (9)	Cl1—Zn1—Cl2	131.14 (4)

**Table 4:** Hydrogen-bond geometry (Å, °) for **II** [Chem scheme1] C*g*3 is the centroid of the pyrazine ring N1/N4/C1/C2/C21/C22, and C*g*5 and C*g*7 are the centroids of rings C12–C17 and C32–C37, respectively.

*D*—H⋯*A*	*D*—H	H⋯*A*	*D*⋯*A*	*D*—H⋯*A*
C6—H6⋯C*g*7^i^	0.94	2.88	3.814 (6)	177
C11—H11*B*⋯C*g*5^ii^	0.98	2.90	3.540 (5)	124
C26—H26⋯C*g*3^iii^	0.94	2.95	3.544 (5)	122
Zn1—Cl2⋯C*g*3^iv^	2.24 (1)	3.68 (1)	5.8035 (19)	156 (1)

Symmetry codes: (i) 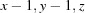; (ii) 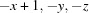; (iii) 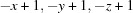; (iv) 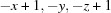.

**Table 5:** A comparison of the conformation of the ligand (**L**) in complexes **I** and **II** The definitions of rings *A*, *B*, *C*, *D* and *E* are given in Fig. 4 ▸.

Dihedral angle (°)	**I** ^*a*^	**II**	Δ(**I - II**)°
*A* to *B*	41.9 (2)	35.5 (2)	> 6.4
*A* to *C*	86.1 (2)	87.5 (3)	< 1.4
*A* to *D*	41.9 (2)	34.9 (2)	> 7.0
*A* to *E*	86.1 (2)	74.4 (2)	> 11.7
*B* to *C*	54.0 (2)	53.7 (3)	> 0.3
*B* to *D*	38.0 (2)	26.9 (2)	> 11.1
*B* to *E*	63.4 (2)	71.9 (2)	< 8.5
*C* to *D*	63.4 (2)	58.5 (3)	> 4.9
*C* to *E*	24.9 (2)	18.3 (3)	> 6.6
*D* to *E*	54.0 (2)	74.9 (2)	< 20.9

Note: (*a*) *D* = *B* ^i^, *E* = *C* ^i^; symmetry code: (i) −*x* + 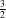, *y*, −*z*.

**Table 6:** Summary of inter­atomic contacts (Å)^*a*^, shorter than the sum of the van der Waals radii, in the crystal structures of **I** and **II**

Contact	Length	Length − vdW	Symmetry operation
**I**			
C11⋯C12	3.278	−0.122	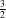 − *x*, − 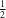 − *y*, − 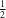 − *z*
C12⋯H11*A*	2.805	−0.095	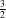 − *x*, − 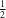 − *y*, − 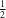 − *z*
I1⋯H17	3.087	−0.093	− 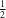 + *x*, −*y*, *z*
N3⋯H11*B*	2.671	−0.079	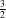 − *x*, − 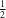 − *y*, − 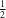 − *z*
N3⋯C11	3.234	−0.016	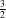 − *x*, − 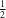 − *y*, − 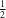 − *z*
			
**II**			
Cl4⋯Cl4	3.045	−0.455	-*x*, −*y*, 2 − *z*
C6⋯H40*B*	2.758	−0.142	-*x*, −*y*, 1 − *z*
C30⋯H3*B*	2.779	−0.121	1 − *x*, −*y*, 1 − *z*
H23*B*⋯H23*B*	2.287	−0.113	1 − *x*, 1 − *y*, 1 − *z*
H6⋯C36	2.798	−0.102	−1 + *x*, −1 + *y*, *z*
Cl1⋯H33	2.854	−0.096	−1 + *x*, *y*, *z*
H6⋯C37	2.858	−0.042	−1 + *x*, −1 + *y*, *z*
H3*B*⋯H30*A*	2.359	−0.041	1 − *x*, −*y*, 1 − *z*
H10*B*⋯H26	2.382	−0.018	1 − *x*, 1 − *y*, 1 − *z*

Note: (*a*) distances were calculated using *Mercury* (Macrae *et al.*, 2008 ▸).

**Table 7:** Experimental details

	**I**	**II**
Crystal data		
Chemical formula	[CdI_2_(C_36_H_40_N_6_)]	[ZnCl_2_(C_36_H_40_N_6_)]·0.6CH_2_Cl_2_
*M*_r_	922.94	743.99
Crystal system, space group	Monoclinic, *I*2/*a*	Triclinic, *P* 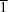
Temperature (K)	223	223
*a*, *b*, *c* (Å)	12.8370 (7), 20.1241 (14), 15.2568 (9)	11.9196 (8), 12.1208 (8), 13.919 (1)
α, β, γ (°)	90, 110.871 (6), 90	98.222 (8), 100.313 (8), 107.580 (7)
*V* (Å^3^)	3682.7 (4)	1843.9 (2)
*Z*	4	2
Radiation type	Mo *K*α	Mo *K*α
μ (mm^−1^)	2.30	0.93
Crystal size (mm)	0.40 × 0.10 × 0.10	0.30 × 0.10 × 0.10

Data collection		
Diffractometer	STOE *IPDS* 1	STOE *IPDS* 1
Absorption correction	Multi-scan (*MULABS*; Spek, 2020 ▸)	Multi-scan (*MULABS*; Spek, 2020 ▸)
*T*_min_, *T*_max_	0.961, 1.000	0.983, 1.000
No. of measured, independent and observed [*I* > 2σ(*I*)] reflections	14308, 3566, 2549	14512, 6654, 3490
*R*_int_	0.031	0.054
(sin θ/λ)_max_ (Å^−1^)	0.615	0.615

Refinement		
*R*[*F* ^2^ > 2σ(*F* ^2^)], *wR*(*F* ^2^), *S*	0.029, 0.072, 0.95	0.043, 0.117, 0.79
No. of reflections	3566	6654
No. of parameters	207	437
H-atom treatment	H-atom parameters constrained	H-atom parameters constrained
Δρ_max_, Δρ_min_ (e Å^−3^)	1.15, −0.88	0.75, −0.35

Computer programs: *EXPOSE*, *CELL* and *INTEGRATE* in *IPDS*-I (Stoe & Cie, 2004 ▸), *SHELXS97* (Sheldrick, 2008 ▸), *Mercury* (Macrae *et al.*, 2020 ▸), *SHELXL2018/3* (Sheldrick, 2015 ▸), *PLATON* (Spek, 2020 ▸) and *publCIF* (Westrip, 2010 ▸).

## supplementary crystallographic information

### Diiodido{N,N',N'',N'''-[pyrazine-2,3,5,6-tetrayltetrakis(methylene)]tetrakis(N-methylaniline)-κ3N2,N1,N6}cadmium(II) (I) Crystal data

**Table:**

[CdI_2_(C_36_H_40_N_6_)]	*F*(000) = 1808
*M**_r_* = 922.94	*D*_x_ = 1.665 Mg m^−^^3^
Monoclinic, *I*2/*a*	Mo *K*α radiation, λ = 0.71073 Å
*a* = 12.8370 (7) Å	Cell parameters from 5000 reflections
*b* = 20.1241 (14) Å	θ = 1.7–26.1°
*c* = 15.2568 (9) Å	µ = 2.30 mm^−^^1^
β = 110.871 (6)°	*T* = 223 K
*V* = 3682.7 (4) Å^3^	Rod, green
*Z* = 4	0.40 × 0.10 × 0.10 mm

### Diiodido{N,N',N'',N'''-[pyrazine-2,3,5,6-tetrayltetrakis(methylene)]tetrakis(N-methylaniline)-κ3N2,N1,N6}cadmium(II) (I) Data collection

**Table:**

STOE IPDS 1 diffractometer	3566 independent reflections
Radiation source: fine-focus sealed tube	2549 reflections with *I* > 2σ(*I*)
Plane graphite monochromator	*R*_int_ = 0.031
φ rotation scans	θ_max_ = 25.9°, θ_min_ = 2.0°
Absorption correction: multi-scan (MULABS; Spek, 2009)	*h* = −15→15
*T*_min_ = 0.961, *T*_max_ = 1.000	*k* = −24→24
14308 measured reflections	*l* = −18→18

### Diiodido{N,N',N'',N'''-[pyrazine-2,3,5,6-tetrayltetrakis(methylene)]tetrakis(N-methylaniline)-κ3N2,N1,N6}cadmium(II) (I) Refinement

**Table:**

Refinement on *F*^2^	Primary atom site location: structure-invariant direct methods
Least-squares matrix: full	Secondary atom site location: difference Fourier map
*R*[*F*^2^ > 2σ(*F*^2^)] = 0.029	Hydrogen site location: inferred from neighbouring sites
*wR*(*F*^2^) = 0.072	H-atom parameters constrained
*S* = 0.95	*w* = 1/[σ^2^(*F*_o_^2^) + (0.0409*P*)^2^] where *P* = (*F*_o_^2^ + 2*F*_c_^2^)/3
3566 reflections	(Δ/σ)_max_ = 0.001
207 parameters	Δρ_max_ = 1.15 e Å^−^^3^
0 restraints	Δρ_min_ = −0.88 e Å^−^^3^

### Diiodido{N,N',N'',N'''-[pyrazine-2,3,5,6-tetrayltetrakis(methylene)]tetrakis(N-methylaniline)-κ3N2,N1,N6}cadmium(II) (I) Special details

**Table:**

Geometry. All esds (except the esd in the dihedral angle between two l.s. planes) are estimated using the full covariance matrix. The cell esds are taken into account individually in the estimation of esds in distances, angles and torsion angles; correlations between esds in cell parameters are only used when they are defined by crystal symmetry. An approximate (isotropic) treatment of cell esds is used for estimating esds involving l.s. planes.

### Diiodido{N,N',N'',N'''-[pyrazine-2,3,5,6-tetrayltetrakis(methylene)]tetrakis(N-methylaniline)-κ3N2,N1,N6}cadmium(II) (I) Fractional atomic coordinates and isotropic or equivalent isotropic displacement parameters (Å^2^)

**Table:**

	*x*	*y*	*z*	*U*_iso_*/*U*_eq_	
Cd1	0.750000	0.06345 (2)	0.000000	0.04883 (11)	
I1	0.55787 (2)	0.11927 (2)	−0.00131 (2)	0.07922 (12)	
N1	0.750000	−0.05058 (17)	0.000000	0.0399 (8)	
N2	0.6906 (2)	0.01854 (14)	−0.17053 (17)	0.0460 (6)	
N3	0.7067 (3)	−0.26232 (15)	−0.15731 (19)	0.0545 (7)	
N4	0.750000	−0.18660 (18)	0.000000	0.0423 (8)	
C1	0.7472 (2)	−0.08374 (16)	−0.0768 (2)	0.0410 (7)	
C2	0.7432 (3)	−0.15288 (16)	−0.0772 (2)	0.0417 (7)	
C3	0.7572 (3)	−0.04292 (16)	−0.1562 (2)	0.0476 (8)	
H3A	0.835630	−0.031459	−0.142427	0.057*	
H3B	0.731715	−0.069302	−0.213986	0.057*	
C4	0.7161 (3)	0.06601 (18)	−0.2319 (2)	0.0513 (8)	
C5	0.7873 (3)	0.0528 (2)	−0.2792 (3)	0.0655 (10)	
H5	0.820979	0.010810	−0.273599	0.079*	
C6	0.8096 (4)	0.1012 (2)	−0.3351 (3)	0.0838 (14)	
H6	0.858370	0.091568	−0.367010	0.101*	
C7	0.7617 (4)	0.1625 (3)	−0.3441 (4)	0.0881 (14)	
H7	0.777532	0.195179	−0.381596	0.106*	
C8	0.6897 (4)	0.1759 (2)	−0.2974 (3)	0.0835 (13)	
H8	0.655115	0.217760	−0.304226	0.100*	
C9	0.6677 (3)	0.1284 (2)	−0.2406 (3)	0.0674 (11)	
H9	0.619914	0.138442	−0.207941	0.081*	
C10	0.5702 (3)	0.0017 (2)	−0.2061 (2)	0.0591 (9)	
H10A	0.550377	−0.019190	−0.267217	0.089*	
H10B	0.526719	0.041903	−0.211542	0.089*	
H10C	0.554820	−0.028697	−0.162881	0.089*	
C11	0.7299 (3)	−0.19289 (17)	−0.1643 (2)	0.0528 (8)	
H11A	0.669076	−0.173622	−0.217143	0.063*	
H11B	0.798405	−0.188947	−0.178391	0.063*	
C12	0.7924 (3)	−0.30691 (18)	−0.1149 (2)	0.0564 (9)	
C13	0.7708 (5)	−0.3737 (2)	−0.1026 (3)	0.0763 (13)	
H13	0.696946	−0.389104	−0.121410	0.092*	
C14	0.8600 (6)	−0.4174 (2)	−0.0619 (4)	0.0975 (17)	
H14	0.844606	−0.462330	−0.054507	0.117*	
C15	0.9680 (6)	−0.3971 (3)	−0.0330 (4)	0.1016 (18)	
H15	1.026535	−0.427029	−0.004708	0.122*	
C16	0.9892 (4)	−0.3321 (3)	−0.0459 (3)	0.0862 (14)	
H16	1.063444	−0.317367	−0.027305	0.103*	
C17	0.9041 (4)	−0.2880 (2)	−0.0856 (3)	0.0664 (10)	
H17	0.921527	−0.243502	−0.093253	0.080*	
C18	0.5935 (4)	−0.2794 (2)	−0.1693 (3)	0.0777 (12)	
H18A	0.569896	−0.317068	−0.211386	0.117*	
H18B	0.545221	−0.241751	−0.195689	0.117*	
H18C	0.589042	−0.290749	−0.108965	0.117*	

### Diiodido{N,N',N'',N'''-[pyrazine-2,3,5,6-tetrayltetrakis(methylene)]tetrakis(N-methylaniline)-κ3N2,N1,N6}cadmium(II) (I) Atomic displacement parameters (Å^2^)

**Table:**

	*U*^11^	*U*^22^	*U*^33^	*U*^12^	*U*^13^	*U*^23^
Cd1	0.03359 (17)	0.0483 (2)	0.0628 (2)	0.000	0.01500 (15)	0.000
I1	0.04735 (16)	0.0918 (2)	0.0946 (2)	0.02423 (13)	0.02050 (14)	−0.00291 (16)
N1	0.0377 (19)	0.043 (2)	0.0401 (19)	0.000	0.0151 (15)	0.000
N2	0.0346 (13)	0.0559 (16)	0.0469 (14)	0.0013 (12)	0.0136 (11)	0.0099 (12)
N3	0.0613 (18)	0.0577 (18)	0.0493 (16)	−0.0119 (15)	0.0254 (14)	−0.0097 (14)
N4	0.049 (2)	0.044 (2)	0.0371 (19)	0.000	0.0191 (16)	0.000
C1	0.0357 (15)	0.0541 (19)	0.0358 (15)	0.0012 (14)	0.0157 (12)	0.0024 (14)
C2	0.0398 (16)	0.0521 (19)	0.0373 (16)	0.0012 (14)	0.0187 (13)	−0.0003 (14)
C3	0.0478 (18)	0.0541 (19)	0.0444 (17)	0.0018 (15)	0.0209 (14)	0.0078 (14)
C4	0.0404 (17)	0.059 (2)	0.0513 (18)	−0.0002 (15)	0.0118 (14)	0.0126 (16)
C5	0.057 (2)	0.076 (3)	0.071 (2)	0.0077 (19)	0.0325 (19)	0.024 (2)
C6	0.074 (3)	0.098 (3)	0.093 (3)	0.013 (3)	0.046 (3)	0.040 (3)
C7	0.075 (3)	0.094 (3)	0.099 (3)	0.003 (3)	0.036 (3)	0.045 (3)
C8	0.071 (3)	0.070 (3)	0.104 (3)	0.010 (2)	0.025 (3)	0.033 (3)
C9	0.052 (2)	0.068 (2)	0.081 (3)	0.0093 (18)	0.023 (2)	0.021 (2)
C10	0.0371 (18)	0.077 (3)	0.058 (2)	−0.0061 (17)	0.0104 (15)	0.0104 (18)
C11	0.068 (2)	0.056 (2)	0.0398 (17)	−0.0015 (17)	0.0263 (16)	−0.0037 (15)
C12	0.080 (3)	0.055 (2)	0.0427 (18)	−0.0036 (19)	0.0322 (18)	−0.0075 (15)
C13	0.113 (4)	0.060 (3)	0.064 (2)	−0.011 (2)	0.041 (3)	−0.009 (2)
C14	0.161 (6)	0.059 (3)	0.084 (3)	0.018 (3)	0.058 (4)	0.005 (2)
C15	0.126 (5)	0.101 (5)	0.088 (4)	0.042 (4)	0.051 (4)	0.004 (3)
C16	0.084 (3)	0.107 (4)	0.074 (3)	0.021 (3)	0.035 (2)	−0.008 (3)
C17	0.076 (3)	0.072 (3)	0.058 (2)	0.002 (2)	0.032 (2)	−0.0091 (19)
C18	0.070 (3)	0.094 (3)	0.072 (3)	−0.023 (2)	0.029 (2)	−0.011 (2)

### Diiodido{N,N',N'',N'''-[pyrazine-2,3,5,6-tetrayltetrakis(methylene)]tetrakis(N-methylaniline)-κ3N2,N1,N6}cadmium(II) (I) Geometric parameters (Å, º)

**Table:**

Cd1—N1	2.295 (3)	C6—H6	0.9400
Cd1—N2^i^	2.599 (3)	C7—C8	1.380 (7)
Cd1—N2	2.599 (3)	C7—H7	0.9400
Cd1—I1^i^	2.7038 (3)	C8—C9	1.386 (6)
Cd1—I1	2.7038 (3)	C8—H8	0.9400
N1—C1	1.338 (3)	C9—H9	0.9400
N1—C1^i^	1.338 (3)	C10—H10A	0.9700
N2—C4	1.454 (4)	C10—H10B	0.9700
N2—C3	1.475 (4)	C10—H10C	0.9700
N2—C10	1.484 (4)	C11—H11A	0.9800
N3—C12	1.388 (5)	C11—H11B	0.9800
N3—C11	1.440 (4)	C12—C17	1.394 (6)
N3—C18	1.440 (5)	C12—C13	1.398 (5)
N4—C2	1.335 (4)	C13—C14	1.402 (7)
N4—C2^i^	1.335 (4)	C13—H13	0.9400
C1—C2	1.392 (5)	C14—C15	1.360 (9)
C1—C3	1.506 (4)	C14—H14	0.9400
C2—C11	1.511 (4)	C15—C16	1.364 (8)
C3—H3A	0.9800	C15—H15	0.9400
C3—H3B	0.9800	C16—C17	1.370 (6)
C4—C5	1.376 (5)	C16—H16	0.9400
C4—C9	1.386 (5)	C17—H17	0.9400
C5—C6	1.390 (5)	C18—H18A	0.9700
C5—H5	0.9400	C18—H18B	0.9700
C6—C7	1.363 (7)	C18—H18C	0.9700
			
N1—Cd1—N2^i^	69.65 (6)	C6—C7—H7	120.5
N1—Cd1—N2	69.65 (6)	C8—C7—H7	120.5
N2^i^—Cd1—N2	139.31 (12)	C7—C8—C9	120.7 (4)
N1—Cd1—I1^i^	114.551 (10)	C7—C8—H8	119.6
N2^i^—Cd1—I1^i^	101.29 (6)	C9—C8—H8	119.6
N1—Cd1—I1	114.551 (10)	C8—C9—C4	120.1 (4)
N2^i^—Cd1—I1	95.35 (6)	C8—C9—H9	120.0
N2—Cd1—I1^i^	95.35 (6)	C4—C9—H9	120.0
N2—Cd1—I1	101.29 (6)	N2—C10—H10A	109.5
I1^i^—Cd1—I1	130.90 (2)	N2—C10—H10B	109.5
C1—N1—C1^i^	120.2 (4)	H10A—C10—H10B	109.5
C1—N1—Cd1	119.92 (19)	N2—C10—H10C	109.5
C1^i^—N1—Cd1	119.92 (19)	H10A—C10—H10C	109.5
C4—N2—C3	113.3 (3)	H10B—C10—H10C	109.5
C4—N2—C10	111.0 (2)	N3—C11—C2	114.4 (3)
C3—N2—C10	109.6 (3)	N3—C11—H11A	108.7
C4—N2—Cd1	111.3 (2)	C2—C11—H11A	108.7
C3—N2—Cd1	101.28 (17)	N3—C11—H11B	108.7
C10—N2—Cd1	110.0 (2)	C2—C11—H11B	108.7
C12—N3—C11	120.8 (3)	H11A—C11—H11B	107.6
C12—N3—C18	120.1 (3)	N3—C12—C17	121.8 (3)
C11—N3—C18	116.6 (3)	N3—C12—C13	121.4 (4)
C2—N4—C2^i^	118.9 (4)	C17—C12—C13	116.8 (4)
N1—C1—C2	119.5 (3)	C12—C13—C14	119.6 (5)
N1—C1—C3	116.7 (3)	C12—C13—H13	120.2
C2—C1—C3	123.7 (3)	C14—C13—H13	120.2
N4—C2—C1	120.9 (3)	C15—C14—C13	122.1 (5)
N4—C2—C11	117.2 (3)	C15—C14—H14	118.9
C1—C2—C11	121.9 (3)	C13—C14—H14	118.9
N2—C3—C1	111.4 (3)	C14—C15—C16	118.4 (5)
N2—C3—H3A	109.3	C14—C15—H15	120.8
C1—C3—H3A	109.3	C16—C15—H15	120.8
N2—C3—H3B	109.3	C15—C16—C17	121.0 (5)
C1—C3—H3B	109.3	C15—C16—H16	119.5
H3A—C3—H3B	108.0	C17—C16—H16	119.5
C5—C4—C9	118.9 (3)	C16—C17—C12	122.1 (4)
C5—C4—N2	123.7 (3)	C16—C17—H17	118.9
C9—C4—N2	117.4 (3)	C12—C17—H17	118.9
C4—C5—C6	120.5 (4)	N3—C18—H18A	109.5
C4—C5—H5	119.8	N3—C18—H18B	109.5
C6—C5—H5	119.8	H18A—C18—H18B	109.5
C7—C6—C5	120.7 (4)	N3—C18—H18C	109.5
C7—C6—H6	119.6	H18A—C18—H18C	109.5
C5—C6—H6	119.6	H18B—C18—H18C	109.5
C6—C7—C8	119.1 (4)		
			
C1^i^—N1—C1—C2	2.3 (2)	C4—C5—C6—C7	0.0 (7)
Cd1—N1—C1—C2	−177.7 (2)	C5—C6—C7—C8	−0.5 (8)
C1^i^—N1—C1—C3	−173.3 (3)	C6—C7—C8—C9	1.2 (7)
Cd1—N1—C1—C3	6.7 (3)	C7—C8—C9—C4	−1.6 (7)
C2^i^—N4—C2—C1	2.3 (2)	C5—C4—C9—C8	1.1 (6)
C2^i^—N4—C2—C11	−177.0 (3)	N2—C4—C9—C8	179.4 (4)
N1—C1—C2—N4	−4.7 (4)	C12—N3—C11—C2	−82.7 (4)
C3—C1—C2—N4	170.6 (3)	C18—N3—C11—C2	79.2 (4)
N1—C1—C2—C11	174.6 (3)	N4—C2—C11—N3	10.3 (4)
C3—C1—C2—C11	−10.1 (5)	C1—C2—C11—N3	−169.0 (3)
C4—N2—C3—C1	167.5 (3)	C11—N3—C12—C17	−6.2 (5)
C10—N2—C3—C1	−68.0 (3)	C18—N3—C12—C17	−167.4 (3)
Cd1—N2—C3—C1	48.2 (3)	C11—N3—C12—C13	176.2 (3)
N1—C1—C3—N2	−41.9 (4)	C18—N3—C12—C13	15.0 (5)
C2—C1—C3—N2	142.8 (3)	N3—C12—C13—C14	178.1 (4)
C3—N2—C4—C5	5.8 (4)	C17—C12—C13—C14	0.3 (6)
C10—N2—C4—C5	−118.0 (4)	C12—C13—C14—C15	0.6 (7)
Cd1—N2—C4—C5	119.1 (3)	C13—C14—C15—C16	−1.3 (8)
C3—N2—C4—C9	−172.4 (3)	C14—C15—C16—C17	1.2 (8)
C10—N2—C4—C9	63.8 (4)	C15—C16—C17—C12	−0.3 (7)
Cd1—N2—C4—C9	−59.1 (3)	N3—C12—C17—C16	−178.2 (3)
C9—C4—C5—C6	−0.3 (6)	C13—C12—C17—C16	−0.5 (5)
N2—C4—C5—C6	−178.5 (4)		

Symmetry code: (i) −*x*+3/2, *y*, −*z*.

### Diiodido{N,N',N'',N'''-[pyrazine-2,3,5,6-tetrayltetrakis(methylene)]tetrakis(N-methylaniline)-κ3N2,N1,N6}cadmium(II) (I) Hydrogen-bond geometry (Å, º)

C*g*3 is the centroid of the pyrazine ring N1/N4/C1/C2/C1^i^/C2^i^ and C*g*5 is the centroid of the C12–C17 ring.

**Table:**

*D*—H···*A*	*D*—H	H···*A*	*D*···*A*	*D*—H···*A*
C18—H18*C*···C*g*5^i^	0.97	2.95	3.896 (5)	165
C17—H17···I1^ii^	0.94	3.09	3.907 (4)	147
Cd1—I1···C*g*^iii^	2.70 (1)	3.96 (1)	6.5131 (12)	155 (1)
Cd1—I1···C*g*3^iv^	2.70 (1)	3.96 (1)	6.5131 (12)	155 (1)

Symmetry codes: (i) −*x*+3/2, *y*, −*z*; (ii) *x*+1/2, −*y*, *z*; (iii) −*x*+1, −*y*, −*z*; (iv) *x*−1/2, −*y*, *z*.

### Dichorido{N,N',N'',N'''-[pyrazine-2,3,5,6-tetrayltetrakis(methylene)]tetrakis(N-methylaniline)-κ3N2,N1,N6}zinc(II) dichloromethane 0.6-solvate (II) Crystal data

**Table:**

[ZnCl_2_(C_36_H_40_N_6_)]·0.6CH_2_Cl_2_	*Z* = 2
*M**_r_* = 743.99	*F*(000) = 774.4
Triclinic, *P*1	*D*_x_ = 1.340 Mg m^−^^3^
*a* = 11.9196 (8) Å	Mo *K*α radiation, λ = 0.71073 Å
*b* = 12.1208 (8) Å	Cell parameters from 5000 reflections
*c* = 13.919 (1) Å	θ = 1.7–26.1°
α = 98.222 (8)°	µ = 0.93 mm^−^^1^
β = 100.313 (8)°	*T* = 223 K
γ = 107.580 (7)°	Rod, yellow
*V* = 1843.9 (2) Å^3^	0.30 × 0.10 × 0.10 mm

### Dichorido{N,N',N'',N'''-[pyrazine-2,3,5,6-tetrayltetrakis(methylene)]tetrakis(N-methylaniline)-κ3N2,N1,N6}zinc(II) dichloromethane 0.6-solvate (II) Data collection

**Table:**

STOE IPDS 1 diffractometer	6654 independent reflections
Radiation source: fine-focus sealed tube	3490 reflections with *I* > 2σ(*I*)
Plane graphite monochromator	*R*_int_ = 0.054
φ rotation scans	θ_max_ = 25.9°, θ_min_ = 2.1°
Absorption correction: multi-scan (MULABS; Spek, 2009)	*h* = −14→13
*T*_min_ = 0.983, *T*_max_ = 1.000	*k* = −13→14
14512 measured reflections	*l* = −17→17

### Dichorido{N,N',N'',N'''-[pyrazine-2,3,5,6-tetrayltetrakis(methylene)]tetrakis(N-methylaniline)-κ3N2,N1,N6}zinc(II) dichloromethane 0.6-solvate (II) Refinement

**Table:**

Refinement on *F*^2^	Primary atom site location: structure-invariant direct methods
Least-squares matrix: full	Secondary atom site location: difference Fourier map
*R*[*F*^2^ > 2σ(*F*^2^)] = 0.043	Hydrogen site location: inferred from neighbouring sites
*wR*(*F*^2^) = 0.117	H-atom parameters constrained
*S* = 0.79	*w* = 1/[σ^2^(*F*_o_^2^) + (0.0649*P*)^2^] where *P* = (*F*_o_^2^ + 2*F*_c_^2^)/3
6654 reflections	(Δ/σ)_max_ = 0.028
437 parameters	Δρ_max_ = 0.75 e Å^−^^3^
0 restraints	Δρ_min_ = −0.35 e Å^−^^3^

### Dichorido{N,N',N'',N'''-[pyrazine-2,3,5,6-tetrayltetrakis(methylene)]tetrakis(N-methylaniline)-κ3N2,N1,N6}zinc(II) dichloromethane 0.6-solvate (II) Special details

**Table:**

Geometry. All esds (except the esd in the dihedral angle between two l.s. planes) are estimated using the full covariance matrix. The cell esds are taken into account individually in the estimation of esds in distances, angles and torsion angles; correlations between esds in cell parameters are only used when they are defined by crystal symmetry. An approximate (isotropic) treatment of cell esds is used for estimating esds involving l.s. planes.

### Dichorido{N,N',N'',N'''-[pyrazine-2,3,5,6-tetrayltetrakis(methylene)]tetrakis(N-methylaniline)-κ3N2,N1,N6}zinc(II) dichloromethane 0.6-solvate (II) Fractional atomic coordinates and isotropic or equivalent isotropic displacement parameters (Å^2^)

**Table:**

	*x*	*y*	*z*	*U*_iso_*/*U*_eq_	Occ. (<1)
Zn1	0.35680 (4)	0.17960 (5)	0.48236 (4)	0.03370 (16)	
Cl1	0.26266 (9)	0.31262 (9)	0.48265 (8)	0.0379 (3)	
Cl2	0.33732 (10)	0.02609 (9)	0.55847 (8)	0.0383 (3)	
N1	0.4877 (3)	0.2055 (3)	0.4016 (2)	0.0294 (8)	
N2	0.2552 (3)	0.0630 (3)	0.3191 (2)	0.0354 (8)	
N3	0.6536 (3)	0.0643 (4)	0.1349 (3)	0.0463 (10)	
N4	0.6673 (3)	0.2247 (3)	0.3005 (2)	0.0353 (9)	
N5	0.5392 (3)	0.3173 (3)	0.5955 (2)	0.0297 (8)	
N6	0.9012 (3)	0.3813 (4)	0.3867 (3)	0.0440 (10)	
C1	0.4709 (4)	0.1232 (4)	0.3201 (3)	0.0309 (10)	
C2	0.5649 (4)	0.1337 (4)	0.2698 (3)	0.0318 (10)	
C3	0.3531 (4)	0.0225 (4)	0.2939 (3)	0.0370 (10)	
H3A	0.332467	−0.012157	0.222331	0.044*	
H3B	0.361523	−0.039048	0.330527	0.044*	
C4	0.1483 (4)	−0.0309 (4)	0.3234 (3)	0.0380 (11)	
C5	0.1318 (4)	−0.1506 (4)	0.2941 (3)	0.0440 (11)	
H5	0.189961	−0.173802	0.267030	0.053*	
C6	0.0295 (4)	−0.2354 (5)	0.3049 (3)	0.0524 (13)	
H6	0.020101	−0.315972	0.286843	0.063*	
C7	−0.0586 (5)	−0.2035 (6)	0.3417 (4)	0.0615 (15)	
H7	−0.128003	−0.261909	0.348052	0.074*	
C8	−0.0441 (4)	−0.0849 (6)	0.3691 (4)	0.0618 (15)	
H8	−0.104135	−0.062684	0.393942	0.074*	
C9	0.0583 (4)	0.0012 (5)	0.3603 (4)	0.0518 (13)	
H9	0.067382	0.081656	0.379134	0.062*	
C10	0.2243 (4)	0.1374 (4)	0.2491 (3)	0.0492 (12)	
H10C	0.195802	0.090689	0.181450	0.074*	
H10B	0.295684	0.204609	0.253252	0.074*	
H10A	0.161260	0.165493	0.267231	0.074*	
C11	0.5545 (4)	0.0370 (4)	0.1832 (3)	0.0458 (12)	
H11A	0.549429	−0.035832	0.207651	0.055*	
H11B	0.479057	0.021761	0.133629	0.055*	
C12	0.6568 (4)	0.1362 (4)	0.0662 (3)	0.0447 (12)	
C13	0.7525 (5)	0.1612 (5)	0.0174 (4)	0.0666 (16)	
H13	0.814974	0.129703	0.032399	0.080*	
C14	0.7552 (7)	0.2312 (7)	−0.0519 (5)	0.087 (2)	
H14	0.820382	0.246888	−0.083057	0.105*	
C15	0.6682 (9)	0.2781 (6)	−0.0769 (5)	0.098 (3)	
H15	0.671980	0.325867	−0.124647	0.117*	
C16	0.5726 (7)	0.2540 (6)	−0.0303 (4)	0.086 (2)	
H16	0.510787	0.285978	−0.046768	0.103*	
C17	0.5667 (5)	0.1833 (5)	0.0405 (4)	0.0599 (14)	
H17	0.500786	0.167545	0.070873	0.072*	
C18	0.7620 (6)	0.0419 (6)	0.1793 (4)	0.086 (2)	
H18C	0.779738	−0.012043	0.130429	0.129*	
H18B	0.749440	0.006775	0.236560	0.129*	
H18A	0.829462	0.115809	0.200454	0.129*	
C21	0.5910 (4)	0.2969 (4)	0.4341 (3)	0.0288 (9)	
C22	0.6828 (3)	0.3080 (4)	0.3814 (3)	0.0310 (10)	
C23	0.5972 (4)	0.3833 (4)	0.5261 (3)	0.0324 (10)	
H23A	0.681896	0.429429	0.558643	0.039*	
H23B	0.555559	0.438161	0.507247	0.039*	
C24	0.5072 (4)	0.3885 (4)	0.6722 (3)	0.0317 (10)	
C25	0.5468 (4)	0.5100 (4)	0.6919 (3)	0.0362 (10)	
H25	0.599552	0.551869	0.656583	0.043*	
C26	0.5080 (4)	0.5711 (4)	0.7651 (3)	0.0448 (12)	
H26	0.533850	0.654230	0.778053	0.054*	
C27	0.4327 (4)	0.5106 (5)	0.8179 (3)	0.0487 (13)	
H27	0.407923	0.552419	0.867503	0.058*	
C28	0.3934 (4)	0.3893 (5)	0.7988 (3)	0.0489 (12)	
H28	0.341885	0.347757	0.835120	0.059*	
C29	0.4302 (4)	0.3282 (4)	0.7255 (3)	0.0440 (12)	
H29	0.402641	0.245046	0.711871	0.053*	
C30	0.6175 (4)	0.2547 (4)	0.6402 (3)	0.0432 (12)	
H30C	0.693683	0.312083	0.679612	0.065*	
H30B	0.632752	0.203586	0.587463	0.065*	
H30A	0.577342	0.207377	0.682678	0.065*	
C31	0.7994 (4)	0.4100 (4)	0.4112 (3)	0.0463 (12)	
H31A	0.788908	0.473627	0.378260	0.056*	
H31B	0.817746	0.440047	0.483454	0.056*	
C32	0.9419 (4)	0.4069 (4)	0.3029 (3)	0.0368 (10)	
C33	1.0564 (4)	0.4040 (5)	0.2933 (3)	0.0509 (13)	
H33	1.103887	0.380026	0.342028	0.061*	
C34	1.0987 (5)	0.4367 (6)	0.2120 (4)	0.0689 (17)	
H34	1.175749	0.434902	0.207024	0.083*	
C35	1.0337 (5)	0.4715 (5)	0.1385 (4)	0.0710 (17)	
H35	1.065545	0.495398	0.084821	0.085*	
C36	0.9177 (5)	0.4703 (5)	0.1459 (4)	0.0608 (14)	
H36	0.869357	0.490972	0.095273	0.073*	
C37	0.8741 (4)	0.4395 (4)	0.2261 (3)	0.0480 (12)	
H37	0.796333	0.440162	0.229773	0.058*	
C38	0.9460 (5)	0.3087 (5)	0.4473 (4)	0.0611 (15)	
H38C	0.951629	0.241231	0.404214	0.092*	
H38B	0.890860	0.281192	0.489074	0.092*	
H38A	1.025495	0.355234	0.489222	0.092*	
C40	0.1063 (10)	0.2516 (11)	0.9543 (8)	0.095 (4)	0.6
H40A	0.053445	0.294842	0.973082	0.114*	0.6
H40B	0.103192	0.249458	0.883154	0.114*	0.6
Cl3	0.2564 (2)	0.3346 (3)	1.02194 (16)	0.0912 (10)	0.6
Cl4	0.0491 (3)	0.1163 (3)	0.9673 (2)	0.1003 (10)	0.6

### Dichorido{N,N',N'',N'''-[pyrazine-2,3,5,6-tetrayltetrakis(methylene)]tetrakis(N-methylaniline)-κ3N2,N1,N6}zinc(II) dichloromethane 0.6-solvate (II) Atomic displacement parameters (Å^2^)

**Table:**

	*U*^11^	*U*^22^	*U*^33^	*U*^12^	*U*^13^	*U*^23^
Zn1	0.0365 (3)	0.0345 (3)	0.0385 (3)	0.0163 (3)	0.0187 (2)	0.0121 (2)
Cl1	0.0334 (6)	0.0367 (7)	0.0497 (6)	0.0193 (6)	0.0121 (5)	0.0092 (5)
Cl2	0.0485 (7)	0.0319 (6)	0.0394 (6)	0.0134 (6)	0.0156 (5)	0.0167 (5)
N1	0.032 (2)	0.031 (2)	0.0280 (18)	0.0121 (19)	0.0103 (15)	0.0056 (15)
N2	0.032 (2)	0.033 (2)	0.044 (2)	0.0111 (19)	0.0145 (16)	0.0112 (16)
N3	0.052 (2)	0.052 (3)	0.043 (2)	0.023 (2)	0.0239 (19)	0.0053 (19)
N4	0.037 (2)	0.035 (2)	0.036 (2)	0.011 (2)	0.0168 (16)	0.0051 (17)
N5	0.037 (2)	0.029 (2)	0.0307 (18)	0.0164 (18)	0.0140 (15)	0.0092 (15)
N6	0.034 (2)	0.059 (3)	0.046 (2)	0.018 (2)	0.0189 (18)	0.0173 (19)
C1	0.034 (2)	0.028 (3)	0.033 (2)	0.011 (2)	0.0123 (18)	0.0089 (19)
C2	0.037 (3)	0.029 (3)	0.033 (2)	0.013 (2)	0.0150 (19)	0.0061 (18)
C3	0.037 (3)	0.031 (3)	0.042 (3)	0.010 (2)	0.013 (2)	0.003 (2)
C4	0.032 (2)	0.038 (3)	0.040 (2)	0.005 (2)	0.0093 (19)	0.010 (2)
C5	0.044 (3)	0.037 (3)	0.046 (3)	0.007 (3)	0.011 (2)	0.007 (2)
C6	0.050 (3)	0.043 (3)	0.050 (3)	−0.002 (3)	0.006 (2)	0.009 (2)
C7	0.042 (3)	0.071 (5)	0.055 (3)	−0.007 (3)	0.013 (3)	0.015 (3)
C8	0.036 (3)	0.079 (5)	0.063 (3)	0.007 (3)	0.020 (2)	0.006 (3)
C9	0.038 (3)	0.051 (3)	0.064 (3)	0.011 (3)	0.019 (2)	0.003 (3)
C10	0.049 (3)	0.048 (3)	0.049 (3)	0.015 (3)	0.005 (2)	0.019 (2)
C11	0.055 (3)	0.038 (3)	0.044 (3)	0.012 (3)	0.024 (2)	−0.001 (2)
C12	0.052 (3)	0.046 (3)	0.029 (2)	0.011 (3)	0.012 (2)	−0.006 (2)
C13	0.060 (3)	0.078 (5)	0.044 (3)	−0.003 (3)	0.024 (3)	−0.002 (3)
C14	0.090 (5)	0.095 (6)	0.047 (4)	−0.012 (5)	0.026 (4)	0.002 (4)
C15	0.149 (8)	0.071 (5)	0.036 (4)	−0.011 (5)	0.017 (4)	0.005 (3)
C16	0.141 (7)	0.066 (4)	0.038 (3)	0.042 (5)	−0.004 (4)	−0.007 (3)
C17	0.080 (4)	0.058 (4)	0.039 (3)	0.025 (3)	0.014 (3)	−0.001 (2)
C18	0.086 (4)	0.122 (6)	0.081 (4)	0.072 (5)	0.032 (4)	0.023 (4)
C21	0.032 (2)	0.028 (3)	0.028 (2)	0.011 (2)	0.0081 (18)	0.0060 (18)
C22	0.028 (2)	0.032 (3)	0.036 (2)	0.011 (2)	0.0136 (18)	0.0078 (19)
C23	0.033 (2)	0.031 (3)	0.035 (2)	0.011 (2)	0.0126 (19)	0.0058 (19)
C24	0.033 (2)	0.035 (3)	0.028 (2)	0.015 (2)	0.0075 (18)	0.0027 (18)
C25	0.039 (3)	0.038 (3)	0.034 (2)	0.016 (2)	0.0104 (19)	0.006 (2)
C26	0.056 (3)	0.042 (3)	0.038 (3)	0.020 (3)	0.014 (2)	−0.001 (2)
C27	0.054 (3)	0.060 (4)	0.036 (3)	0.029 (3)	0.014 (2)	−0.002 (2)
C28	0.053 (3)	0.060 (4)	0.040 (3)	0.021 (3)	0.023 (2)	0.010 (2)
C29	0.058 (3)	0.040 (3)	0.038 (3)	0.016 (3)	0.023 (2)	0.006 (2)
C30	0.050 (3)	0.050 (3)	0.039 (3)	0.031 (3)	0.009 (2)	0.008 (2)
C31	0.039 (3)	0.046 (3)	0.052 (3)	0.008 (3)	0.025 (2)	−0.001 (2)
C32	0.031 (2)	0.038 (3)	0.036 (2)	0.007 (2)	0.0075 (19)	−0.001 (2)
C33	0.039 (3)	0.076 (4)	0.040 (3)	0.021 (3)	0.015 (2)	0.008 (2)
C34	0.048 (3)	0.105 (5)	0.052 (3)	0.021 (4)	0.024 (3)	0.007 (3)
C35	0.076 (4)	0.092 (5)	0.041 (3)	0.016 (4)	0.027 (3)	0.011 (3)
C36	0.077 (4)	0.051 (4)	0.046 (3)	0.012 (3)	0.008 (3)	0.015 (3)
C37	0.043 (3)	0.045 (3)	0.054 (3)	0.012 (3)	0.011 (2)	0.011 (2)
C38	0.046 (3)	0.082 (4)	0.058 (3)	0.019 (3)	0.013 (3)	0.030 (3)
C40	0.082 (8)	0.114 (10)	0.084 (8)	0.037 (8)	−0.013 (6)	0.038 (7)
Cl3	0.0444 (13)	0.179 (3)	0.0384 (12)	0.0251 (17)	0.0150 (10)	0.0076 (15)
Cl4	0.088 (2)	0.098 (3)	0.115 (2)	0.043 (2)	0.0134 (18)	0.0124 (19)

### Dichorido{N,N',N'',N'''-[pyrazine-2,3,5,6-tetrayltetrakis(methylene)]tetrakis(N-methylaniline)-κ3N2,N1,N6}zinc(II) dichloromethane 0.6-solvate (II) Geometric parameters (Å, º)

**Table:**

Zn1—N1	2.057 (3)	C15—C16	1.386 (10)
Zn1—N2	2.385 (3)	C15—H15	0.9400
Zn1—N5	2.413 (3)	C16—C17	1.394 (8)
Zn1—Cl1	2.2251 (10)	C16—H16	0.9400
Zn1—Cl2	2.2425 (11)	C17—H17	0.9400
N1—C21	1.334 (5)	C18—H18C	0.9700
N1—C1	1.340 (5)	C18—H18B	0.9700
N2—C4	1.446 (5)	C18—H18A	0.9700
N2—C3	1.473 (5)	C21—C22	1.407 (5)
N2—C10	1.491 (5)	C21—C23	1.509 (5)
N3—C12	1.381 (6)	C22—C31	1.497 (6)
N3—C18	1.449 (6)	C23—H23A	0.9800
N3—C11	1.438 (5)	C23—H23B	0.9800
N4—C2	1.325 (5)	C24—C25	1.373 (6)
N4—C22	1.345 (5)	C24—C29	1.386 (6)
N5—C24	1.453 (5)	C25—C26	1.399 (5)
N5—C30	1.480 (5)	C25—H25	0.9400
N5—C23	1.476 (5)	C26—C27	1.371 (7)
N6—C32	1.380 (5)	C26—H26	0.9400
N6—C31	1.441 (5)	C27—C28	1.371 (7)
N6—C38	1.451 (6)	C27—H27	0.9400
C1—C2	1.408 (5)	C28—C29	1.385 (6)
C1—C3	1.500 (6)	C28—H28	0.9400
C2—C11	1.516 (5)	C29—H29	0.9400
C3—H3A	0.9800	C30—H30C	0.9700
C3—H3B	0.9800	C30—H30B	0.9700
C4—C5	1.393 (6)	C30—H30A	0.9700
C4—C9	1.400 (6)	C31—H31A	0.9800
C5—C6	1.385 (6)	C31—H31B	0.9800
C5—H5	0.9400	C32—C37	1.399 (6)
C6—C7	1.376 (7)	C32—C33	1.404 (6)
C6—H6	0.9400	C33—C34	1.380 (7)
C7—C8	1.383 (8)	C33—H33	0.9400
C7—H7	0.9400	C34—C35	1.365 (7)
C8—C9	1.384 (7)	C34—H34	0.9400
C8—H8	0.9400	C35—C36	1.401 (7)
C9—H9	0.9400	C35—H35	0.9400
C10—H10C	0.9700	C36—C37	1.365 (7)
C10—H10B	0.9700	C36—H36	0.9400
C10—H10A	0.9700	C37—H37	0.9400
C11—H11A	0.9800	C38—H38C	0.9700
C11—H11B	0.9800	C38—H38B	0.9700
C12—C17	1.378 (6)	C38—H38A	0.9700
C12—C13	1.408 (7)	C40—Cl4	1.625 (12)
C13—C14	1.370 (9)	C40—Cl3	1.772 (11)
C13—H13	0.9400	C40—H40A	0.9800
C14—C15	1.343 (9)	C40—H40B	0.9800
C14—H14	0.9400		
			
N1—Zn1—N2	75.02 (12)	C16—C15—H15	121.0
N1—Zn1—N5	74.23 (12)	C17—C16—C15	121.2 (6)
N2—Zn1—N5	149.21 (11)	C17—C16—H16	119.4
N1—Zn1—Cl1	114.15 (9)	C15—C16—H16	119.4
N1—Zn1—Cl2	114.68 (9)	C12—C17—C16	120.4 (6)
Cl1—Zn1—N2	98.12 (8)	C12—C17—H17	119.8
Cl2—Zn1—N2	95.70 (9)	C16—C17—H17	119.8
Cl1—Zn1—N5	93.02 (7)	N3—C18—H18C	109.5
Cl2—Zn1—N5	98.34 (8)	N3—C18—H18B	109.5
Cl1—Zn1—Cl2	131.14 (4)	H18C—C18—H18B	109.5
C21—N1—C1	120.8 (3)	N3—C18—H18A	109.5
C21—N1—Zn1	120.7 (2)	H18C—C18—H18A	109.5
C1—N1—Zn1	118.3 (3)	H18B—C18—H18A	109.5
C4—N2—C3	114.5 (3)	N1—C21—C22	119.6 (3)
C4—N2—C10	111.2 (3)	N1—C21—C23	115.2 (3)
C3—N2—C10	109.9 (3)	C22—C21—C23	125.1 (4)
C4—N2—Zn1	110.2 (2)	N4—C22—C21	119.8 (4)
C3—N2—Zn1	99.2 (2)	N4—C22—C31	117.4 (3)
C10—N2—Zn1	111.3 (3)	C21—C22—C31	122.8 (4)
C12—N3—C18	120.4 (4)	N5—C23—C21	109.2 (3)
C12—N3—C11	120.1 (4)	N5—C23—H23A	109.8
C18—N3—C11	117.4 (4)	C21—C23—H23A	109.8
C2—N4—C22	119.8 (3)	N5—C23—H23B	109.8
C24—N5—C30	111.1 (3)	C21—C23—H23B	109.8
C24—N5—C23	114.7 (3)	H23A—C23—H23B	108.3
C30—N5—C23	109.3 (3)	C25—C24—C29	119.5 (4)
C24—N5—Zn1	109.4 (2)	C25—C24—N5	123.5 (4)
C30—N5—Zn1	111.1 (3)	C29—C24—N5	117.0 (4)
C23—N5—Zn1	100.9 (2)	C24—C25—C26	119.4 (4)
C32—N6—C31	122.1 (4)	C24—C25—H25	120.3
C32—N6—C38	122.1 (3)	C26—C25—H25	120.3
C31—N6—C38	115.2 (4)	C27—C26—C25	120.5 (4)
N1—C1—C2	118.9 (4)	C27—C26—H26	119.7
N1—C1—C3	115.6 (3)	C25—C26—H26	119.7
C2—C1—C3	125.4 (4)	C28—C27—C26	120.2 (4)
N4—C2—C1	120.9 (4)	C28—C27—H27	119.9
N4—C2—C11	118.2 (3)	C26—C27—H27	119.9
C1—C2—C11	120.7 (4)	C27—C28—C29	119.6 (4)
N2—C3—C1	110.9 (3)	C27—C28—H28	120.2
N2—C3—H3A	109.5	C29—C28—H28	120.2
C1—C3—H3A	109.5	C28—C29—C24	120.7 (4)
N2—C3—H3B	109.5	C28—C29—H29	119.6
C1—C3—H3B	109.5	C24—C29—H29	119.6
H3A—C3—H3B	108.1	N5—C30—H30C	109.5
C5—C4—C9	118.8 (4)	N5—C30—H30B	109.5
C5—C4—N2	123.3 (4)	H30C—C30—H30B	109.5
C9—C4—N2	117.8 (4)	N5—C30—H30A	109.5
C6—C5—C4	120.0 (4)	H30C—C30—H30A	109.5
C6—C5—H5	120.0	H30B—C30—H30A	109.5
C4—C5—H5	120.0	N6—C31—C22	114.2 (4)
C7—C6—C5	121.1 (5)	N6—C31—H31A	108.7
C7—C6—H6	119.5	C22—C31—H31A	108.7
C5—C6—H6	119.5	N6—C31—H31B	108.7
C6—C7—C8	119.4 (5)	C22—C31—H31B	108.7
C6—C7—H7	120.3	H31A—C31—H31B	107.6
C8—C7—H7	120.3	N6—C32—C37	122.3 (4)
C7—C8—C9	120.5 (5)	N6—C32—C33	120.4 (4)
C7—C8—H8	119.8	C37—C32—C33	117.3 (4)
C9—C8—H8	119.8	C34—C33—C32	119.6 (5)
C8—C9—C4	120.3 (5)	C34—C33—H33	120.2
C8—C9—H9	119.9	C32—C33—H33	120.2
C4—C9—H9	119.9	C35—C34—C33	122.9 (5)
N2—C10—H10C	109.5	C35—C34—H34	118.5
N2—C10—H10B	109.5	C33—C34—H34	118.5
H10C—C10—H10B	109.5	C34—C35—C36	117.6 (5)
N2—C10—H10A	109.5	C34—C35—H35	121.2
H10C—C10—H10A	109.5	C36—C35—H35	121.2
H10B—C10—H10A	109.5	C37—C36—C35	120.6 (5)
N3—C11—C2	114.2 (4)	C37—C36—H36	119.7
N3—C11—H11A	108.7	C35—C36—H36	119.7
C2—C11—H11A	108.7	C36—C37—C32	121.9 (5)
N3—C11—H11B	108.7	C36—C37—H37	119.1
C2—C11—H11B	108.7	C32—C37—H37	119.1
H11A—C11—H11B	107.6	N6—C38—H38C	109.5
C17—C12—N3	122.3 (4)	N6—C38—H38B	109.5
C17—C12—C13	117.4 (5)	H38C—C38—H38B	109.5
N3—C12—C13	120.3 (4)	N6—C38—H38A	109.5
C14—C13—C12	120.5 (6)	H38C—C38—H38A	109.5
C14—C13—H13	119.8	H38B—C38—H38A	109.5
C12—C13—H13	119.8	Cl4—C40—Cl3	118.1 (6)
C15—C14—C13	122.5 (6)	Cl4—C40—H40A	107.8
C15—C14—H14	118.8	Cl3—C40—H40A	107.8
C13—C14—H14	118.8	Cl4—C40—H40B	107.8
C14—C15—C16	118.0 (6)	Cl3—C40—H40B	107.8
C14—C15—H15	121.0	H40A—C40—H40B	107.1
			
C21—N1—C1—C2	0.9 (5)	C1—N1—C21—C22	0.8 (5)
Zn1—N1—C1—C2	−173.8 (3)	Zn1—N1—C21—C22	175.4 (3)
C21—N1—C1—C3	177.6 (3)	C1—N1—C21—C23	179.5 (3)
Zn1—N1—C1—C3	2.8 (4)	Zn1—N1—C21—C23	−5.9 (4)
C22—N4—C2—C1	0.6 (6)	C2—N4—C22—C21	1.2 (6)
C22—N4—C2—C11	−175.4 (4)	C2—N4—C22—C31	−178.7 (4)
N1—C1—C2—N4	−1.7 (6)	N1—C21—C22—N4	−1.9 (5)
C3—C1—C2—N4	−178.0 (4)	C23—C21—C22—N4	179.6 (3)
N1—C1—C2—C11	174.2 (4)	N1—C21—C22—C31	178.0 (4)
C3—C1—C2—C11	−2.0 (6)	C23—C21—C22—C31	−0.5 (6)
C4—N2—C3—C1	−163.3 (3)	C24—N5—C23—C21	−162.9 (3)
C10—N2—C3—C1	70.8 (4)	C30—N5—C23—C21	71.6 (4)
Zn1—N2—C3—C1	−46.0 (3)	Zn1—N5—C23—C21	−45.5 (3)
N1—C1—C3—N2	34.4 (5)	N1—C21—C23—N5	39.1 (4)
C2—C1—C3—N2	−149.2 (4)	C22—C21—C23—N5	−142.4 (4)
C3—N2—C4—C5	−7.8 (6)	C30—N5—C24—C25	112.7 (4)
C10—N2—C4—C5	117.5 (4)	C23—N5—C24—C25	−11.8 (5)
Zn1—N2—C4—C5	−118.6 (4)	Zn1—N5—C24—C25	−124.3 (3)
C3—N2—C4—C9	171.3 (4)	C30—N5—C24—C29	−68.9 (5)
C10—N2—C4—C9	−63.5 (5)	C23—N5—C24—C29	166.6 (3)
Zn1—N2—C4—C9	60.4 (4)	Zn1—N5—C24—C29	54.1 (4)
C9—C4—C5—C6	−2.2 (6)	C29—C24—C25—C26	−0.5 (6)
N2—C4—C5—C6	176.9 (4)	N5—C24—C25—C26	177.9 (4)
C4—C5—C6—C7	1.9 (7)	C24—C25—C26—C27	1.1 (6)
C5—C6—C7—C8	−0.6 (7)	C25—C26—C27—C28	−0.8 (7)
C6—C7—C8—C9	−0.3 (8)	C26—C27—C28—C29	−0.1 (7)
C7—C8—C9—C4	0.0 (8)	C27—C28—C29—C24	0.7 (7)
C5—C4—C9—C8	1.2 (7)	C25—C24—C29—C28	−0.3 (6)
N2—C4—C9—C8	−177.9 (4)	N5—C24—C29—C28	−178.9 (4)
C12—N3—C11—C2	−80.2 (5)	C32—N6—C31—C22	99.2 (5)
C18—N3—C11—C2	83.5 (5)	C38—N6—C31—C22	−72.1 (5)
N4—C2—C11—N3	−8.2 (6)	N4—C22—C31—N6	−30.4 (6)
C1—C2—C11—N3	175.7 (4)	C21—C22—C31—N6	149.7 (4)
C18—N3—C12—C17	−163.8 (5)	C31—N6—C32—C37	−13.3 (7)
C11—N3—C12—C17	−0.6 (7)	C38—N6—C32—C37	157.3 (5)
C18—N3—C12—C13	18.0 (7)	C31—N6—C32—C33	164.9 (4)
C11—N3—C12—C13	−178.8 (4)	C38—N6—C32—C33	−24.5 (7)
C17—C12—C13—C14	0.8 (8)	N6—C32—C33—C34	−176.1 (5)
N3—C12—C13—C14	179.1 (5)	C37—C32—C33—C34	2.2 (7)
C12—C13—C14—C15	−0.4 (10)	C32—C33—C34—C35	−0.5 (9)
C13—C14—C15—C16	0.0 (10)	C33—C34—C35—C36	−1.8 (9)
C14—C15—C16—C17	0.0 (9)	C34—C35—C36—C37	2.3 (9)
N3—C12—C17—C16	−179.1 (5)	C35—C36—C37—C32	−0.7 (8)
C13—C12—C17—C16	−0.8 (7)	N6—C32—C37—C36	176.7 (5)
C15—C16—C17—C12	0.4 (8)	C33—C32—C37—C36	−1.6 (7)

### Dichorido{N,N',N'',N'''-[pyrazine-2,3,5,6-tetrayltetrakis(methylene)]tetrakis(N-methylaniline)-κ3N2,N1,N6}zinc(II) dichloromethane 0.6-solvate (II) Hydrogen-bond geometry (Å, º)

C*g*3 is the centroid of the pyrazine ring N1/N4/C1/C2/C21/C22, and C*g*5 and C*g*7 are the centroids of rings C12–C17 and C32–C37, respectively.

**Table:**

*D*—H···*A*	*D*—H	H···*A*	*D*···*A*	*D*—H···*A*
C6—H6···C*g*7^i^	0.94	2.88	3.814 (6)	177
C11—H11*B*···C*g*5^ii^	0.98	2.90	3.540 (5)	124
C26—H26···C*g*3^iii^	0.94	2.95	3.544 (5)	122
Zn1—Cl2···C*g*3^iv^	2.24 (1)	3.68 (1)	5.8035 (19)	156 (1)

Symmetry codes: (i) *x*−1, *y*−1, *z*; (ii) −*x*+1, −*y*, −*z*; (iii) −*x*+1, −*y*+1, −*z*+1; (iv) −*x*+1, −*y*, −*z*+1.
